# Preoperative hypovitaminosis D and complications in plastic surgery: a pilot study

**DOI:** 10.1590/0100-6991e-20243719-en

**Published:** 2024-06-14

**Authors:** FLÁVIO JOSÉ TEIXEIRA ROCHA ATAÍDE DA MOTTA, IGOR CHAVES GOMES LUNA, ISADORA MOSCARDINI FABIANI, JÚLIA CIBELY DA SILVA SOUZA, VINICYUS EDUARDO MELO AMORIM, JAIRO ZACCHÊ DE SÁ

**Affiliations:** 1 - Universidade Federal de Pernambuco, Serviço de Cirurgia Plástica - Recife - PE - Brasil; 2 - Faculdade de Medicina do ABC, Serviço de Cirurgia Plástica - Santo André - SP - Brasil; 3 - Universidade Federal de Pernambuco, Centro de Ciências Médicas - Recife - PE - Brasil; 4 - Faculdade Pernambucana de Saúde, Curso de Medicina - Recife - PE - Brasil

**Keywords:** Vitamin D, Plastic Surgery, Postoperative Complications, Vitamina D, Cirurgia Plástica, Complicações Pós-operatórias

## Abstract

**Introduction:**

Vitamin D plays a crucial role in various biological processes, including the well-known regulation of the immune system and calcium metabolism. While its involvement in the surgical outcomes of various medical specialties is recognized, there is a lack of consistent data regarding plastic surgery. This study aimed to assess preoperative serum levels of 25-hydroxyvitamin D and its relationship with complications in patients undergoing reconstructive and aesthetic plastic surgeries.

**Methods:**

prospective and observational cohort study, conducted from October 2021 to August 2023 at the Hospital das Clínicas, Universidade Federal de Pernambuco, involving 83 patients.

**Results:**

vitamin D levels were deemed deficient in 7 (8,4%) patients, insufficient in 36 (43,4%), and sufficient in 40 (48,2%). No direct association was demonstrated between deficient or insufficient serum levels of 25-hydroxyvitamin D and the incidence of complications in plastic surgery, even when considering comorbidities.

**Conclusion:**

preoperative hypovitaminosis D was not associated with complications in plastic surgery.

## RESUMO

**Introdução:**

a vitamina D é parte constituinte de diversos processos biológicos. Além da conhecida regulação do sistema imunológico e do metabolismo do cálcio, está envolvida no desfecho cirúrgico de diversas especialidades, porém não há dados consistentes sobre cirurgia plástica. Este estudo teve como objetivo avaliar os níveis séricos pré-operatórios de 25-hidroxivitamina D e sua relação com complicações em pacientes submetidos a cirurgias plásticas reparadoras e estéticas.

**Métodos:**

coorte prospectiva e observacional, conduzida de outubro de 2021 a agosto de 2023 no Hospital das Clínicas da Universidade Federal de Pernambuco, em 83 pacientes.

**Resultados:**

os níveis de vitamina D foram considerados deficientes em 7 (8,4%) dos pacientes, insuficientes em 36 (43,4%) e suficientes em 40 (48,2%). Não se demonstrou associação direta entre níveis séricos deficientes ou insuficientes de 25-hidroxivitamina D e a incidência de complicações em cirurgia plástica, nem mesmo quando associado a comorbidades

**Conclusão:**

a hipovitaminose D pré-operatória não foi associada a complicações em cirurgia plástica.

## INTRODUCTION

Vitamin D or calciferol is an umbrella term for a group of fat-soluble compounds. It has been recognized as an active ingredient since 1919, when its properties on calcium homeostasis and rickets prevention were discovered^1^. It is now known to play an essential role in a wide range of pleiotropic processes, such as modulation of the immune system, regulation of cardiovascular function, influence on the central nervous system, and possible involvement in anti-inflammatory and anti-oncogenic processes^2^ ^,^ ^3^.

Hypovitaminosis D is a problem that affects about one billion people worldwide, reaching up to 36% of adults between 18 and 29 years of age^4^. Data from the National Health and Nutrition Examination Survey (NHANES) in the United States showed that, from 2011 to 2014, the prevalence of Vitamin D levels below 12 ng/mL was 5%, and from 12 to 19 ng/mL, 18%^5^. Among the causes, there is decrease in endogenous synthesis due to low sun exposure or use of sunscreen, decreased intake in institutionalized people and the elderly, malabsorptive conditions such as osteoporosis and celiac disease, and an increase in hepatic catabolism^5^ ^,^ ^6^.

In most cases, patients are asymptomatic but cases of bone pain and tenderness, muscle weakness, and difficulty walking have been described. Calcium, phosphorus, and alkaline phosphatase levels are usually normal, but in chronic cases, hypocalcemia secondary hyperparathyroidism, bone demineralization, and some degree of rickets and osteomalacia occur. Although the active form is 1,25-dihydroxy-vitamin D or calcitriol^7^, 25-hydroxy-vitamin D (25[OH]D) or calcidiol is the best way to measure the vitamin in serum, with levels expressed in nanograms per milliliter (ng/ml)^2^. The Endocrine Society considers sufficiency to be levels above 30 ng/ml, insufficiency between 20 and 30 ng/ml, and deficiency below 20 ng/ml^8^. The appropriate dietary recommendation for individuals up to 70 years of age, as well as pregnant and lactating women, is 600 international units (IU) or 15 micrograms (mcg) daily; after the age of 71, 800 IU or 20 mcg daily is suggested^2^ ^,^ ^9^.

Low serum Vitamin D has been associated with clinical conditions such as myocardial infarction, diabetes, autoimmune diseases, chronic obstructive pulmonary disease, neoplasms, tuberculosis, and increased mortality in the general population^10^. In addition, it has been associated with surgical complications of various natures and specialties. Many of them have a considerable interface with plastic surgery, namely head and neck surgery, hand surgery, digestive system surgery, oncological surgery, mastology, neurosurgery, orthopedics, and urology^11^ ^-^ ^17^. However, there is no solid data associating Vitamin D with complications from reparative and aesthetic procedures. This study aims to fill this gap by optimizing the yet empirical management of surgical patients with hypovitaminosis. 

## METHODS

This is a prospective and observational cohort study, conducted from October 2021 to August 2023, at the Plastic Surgery Service of the Hospital das Clínicas of the Federal University of Pernambuco. It complied with resolution 466/2012 of the National Research Ethics Commission of the National Health Council and with the Declaration of Helsinki. It was initiated only after approval by the Ethics in Research Committee of the institution, under the certificate of presentation for ethical appraisal number 53828321.7.0000.8807.

The study population consisted of consecutive patients operated on at the institution, a total of 83. The inclusion criteria were individuals over 18 years of age, with or without comorbidities, who had undergone reconstructive or aesthetic plastic surgery, and who agreed to participate in the research by signing a informed consent form. Individuals not included were those outside the age range, who did not undergo preoperative serum 25(OH)D measurement, who were not operated on even with dosage, and who refused to participate. Patients who underwent surgery but not dosage, who missed outpatient follow-up, and who chose to leave the study were excluded.

The variables analyzed were sex, age, race, smoking, comorbidities (hypertension, diabetes, and obesity), preoperative serum level of 25(OH)D, surgical site (head and neck: face, chin, nose, ears, and eyelids, including tumors; body contour: abdomen, arms, thighs, and sacral region; and breasts: female and male, including torsoplasty), and complications (minor: outpatient management; major: requiring admissionb and/or new surgery). Epidemiological information was naturally collected in the anamnesis. The 25(OH)D dosage was included in routine preoperative laboratory tests. Throughout the postoperative consultations, we assessed possible surgical complications, whose treatments followed what is already established in the medical literature.

For statistical assessment, we used the Statistical Package for the Social Sciences 25.0 for Windows and Excel 365. All tests were applied with a 95% confidence level. In addition, all results were calculated considering only valid answers, i.e., ignored answers were not included in the statistical analysis. The results were presented in the form of charts and tables, accompanied by their respective absolute and relative frequencies, providing a clear view of the data distributions. For the numerical variables, measures of central tendency and of dispersion were used, allowing a comprehensive description of the characteristics of the variables. To assess associations between categorical variables, were applied the chi-square test and the Fisher’s exact test.

The primary outcome was the preoperative level of 25(OH)D and the secondary outcome was the presence of postoperative complications in patients with insufficient or deficient levels of 25(OH)D.

## RESULTS

Table 1 brings the demographic data regarding sex, race, age, presence and type of comorbidities, and size of surgery.

**Table 1:** Demography.

Variables	n	%
Sex		
Female	65	78,3
Male	18	21,7
Age (years)		
< 60	56	67,5
≥ 60	27	32,5
Race		
Brown	58	70,7
White	24	29,3
Smoking	3	3,61
Comorbidities		
None	35	42,2
Hypertension	19	22,9
Diabetes	11	13,3
Obesity	7	8,4
Surgery		
Head & Neck	32	38,5
Body Contouring	17	20,5
Breasts	34	41

Table 2 shows the 25(OH)D levels, relating age to mean and standard deviation, median, 25^th^ and 75^th^ percentiles, and range.

**Table 2:** 25(OH)D levels.

Variables	n		%
25(OH)D (ng/ml)			
Deficient (≤ 20)	7 36 40		8,4
Insufficient (21-29)			43,4
Sufficient (≥ 30)			48,2
	Mean ± SD	Median (P25; P75)	Range
Age (years)	50.8 ± 16.2	51,0 (39,0; 62,0)	22,0-86,0
25(OH)D (ng/ml)	31.4 ± 13.8	29,4 (23,4; 36,3)	4,3-107,0

SD: standard deviation. P: percentile

Table 3 shows the prevalence of each type of complication. The minor ones did not require readmission or surgical reapproach, as did major ones.

**Table 3:** Minor and major complications.

Variables	n	%
Minor complications		
Yes	22	26,5
No	61	73,5
Type of minor complications		
Hypertrophic scar	3	13,6
Dehiscence	11	50,0
Bruise	2	9,1
Infection	2	9,1
Necrosis	3	13,6
Paresis	1	4,6
Major complications		
Variables	n	%
Yes	6	7,2
No	77	92,8
Type of major complications		
Dehiscence	2	33,3
Infection	1	16,7
Necrosis	2	33,3
Paresis	1	16,7

Table 4 shows the levels of 25(OH)D and their relationship with the surgical sites.

**Table 4:** Surgical sites and 25(OH)D levels.

	25(OH)D (ng/ml)			
Variables	Deficient (≤ 20)	Insufficient (21-29)	Sufficient (≥ 30)	p-value
	n (%)	n (%)	n (%)	
Surgery				
Head & Neck	3 (9,3)	14 (43,75)	15 (46,8)	0,293*
Body Contouring	0 (0,0)	11 (64,7)	6 (35,3)	
Breasts	4 (11,8)	11 (32,4)	19 (55,8)	

(*) Fisher’s exact test.

Table 5 shows the concentrations of 25(OH)D and their relationship with minor and major complications.

**Table 5:** Complications and 25(OH)D levels.

	25(OH)D (ng/ml)			
Variables	Deficient (≤ 20)	Insufficient (21-29)	Sufficient (≥ 30)	p-value
	n (%)	n (%)	n (%)	
Minor complications				
Yes	3 (13,6)	8 (36,4)	11 (50)	0,517*
No	4 (6,6)	28 (45,9)	29 (47,5)	
Type of minor complications				
Hypertrophic scar	0 (0,0)	1 (33,3)	2 (66,7)	0,673**
Dehiscence	1 (9,1)	3 (27,3)	7 (63,6)	
Bruise	0 (0,0)	1 (50,0)	1 (50,0)	
Infection	1 (50,0)	1 (50,0)	0 (0,0)	
Necrosis	1 (33,3)	1 (33,3)	1 (33,3)	
Paresis	0 (0,0)	1 (100,0)	0 (0,0)	
Major complications				
Yes	2 (33,3)	2 (33,3)	2 (33,3)	0,113**
No	5 (6,5)	34 (44,2)	38 (49,3)	
Type of major complications				
Dehiscence	1 (50,0)	1 (50,0)	0 (0,0)	1,000**
Infection	0 (0,0)	0 (0,0)	1 (100,0)	
Necrosis	0 (0,0)	1 (50,0)	1 (50,0)	
Paresis	1 (100,0)	0 (0,0)	0 (0,0)	

(*) Fisher’s exact test; (**) Chi-square test.

We identified no statistically significant associations between any of the variables analyzed and the concentration of 25(OH)D.

## DISCUSSION

Due to the absence of studies demonstrating the association between serum Vitamin D levels and postoperative complications in plastic surgery, it was not possible to previously estimate the number of patients required. Therefore, this exploratory study did not compute a sample size and used a convenient sample, totaling 83 patients. Vitamin D levels were considered deficient in 7 (8.4%) of them, insufficient in 36 (43.4%), and sufficient in 40 (48.2%). The results did not demonstrate a direct association between deficient or insufficient serum 25(OH)D levels and the incidence of complications in plastic surgery, not even when associated with comorbidities.

We believe this to be primarily due to the heterogeneity of the patient population. Differences in sex, age, race, comorbidities, and surgical site may not significantly influence Vitamin D levels, as shown in Table 1. Results may be compromised by the limited sample. Of these, 65 (78.3%) were women. In addition, 58 (70.7%) declared to be brown and the others to be white. The response to the increase in circulating Vitamin D levels after oral supplementation is similar in white and black American women. African-American women have a lower risk of fractures than Caucasian Americans, and African-American men have higher bone density than white or Hispanic American men^6^ ^,^ ^9^.

The association may also be hindered by the large number of risk factors for hypovitaminosis D. Smoking was detected in 3.6% of the population and, among the comorbidities reported, we observed hypertension in 22.9%, diabetes in 13.3%, and obesity in 8.4%. In addition to low sun exposure and intake, the use of corticosteroids and immunosuppression play a role^18^. These factors are more related to surgical complications in plastic surgery than to hypovitaminosis D itself. However, they were not an analysis variable.

Complications were not categorized into groups such as ischemic, infectious, or thrombotic, but followed a pattern similar to that of the Clavien-Dindo classification^19^. The data are shown in Table 5. Those that required only outpatient treatment were considered minor ones, namely, hypertrophic scar, dehiscence, hematoma, infection, paresis, and necrosis. The major ones, i.e., requiring readmission or reoperation, included dehiscence, infection, necrosis, and paresis. On the other hand, it was also not possible to affirm that the vitamin sufficiency could prevent complications.

Collagen formation and remodeling are regulated in part by vitamin C and 25(OH)D, and it is known to be a fundamental component of the healing process, angiogenesis, and lymphangiogenesis. Decreased serum 25(OH)D may lead to poor healing and potential wound dehiscence. Although no statistically significant differences have been documented, morbidly obese patients have an increased risk of Vitamin D and calcium deficiencies due to reduced intestinal absorption and a sedentary lifestyle, with less exposure to sunlight. In addition, obesity is associated with increased sequestration of lipid-soluble vitamins in stored adipose tissue. Even with calcium and Vitamin D supplementation after Roux-en-Y gastric bypass, such patients may present an increase in bone resorption^15^ ^,^ ^20^ ^,^ ^21^. Among the reconstructive surgeries after marked weight loss, circumferential and anchor abdominoplasties, brachioplasties, cruroplasties, and mammoplasties were performed. All of them had punctual dehiscences as minor complications, not requiring therapy other than dressings. Razzaghi et al. and Burkiewicz et al. published placebo-controlled trials in patients with diabetic and venous ulcers, respectively. In the former, there was a reduction in erythema and lesion size after oral Vitamin D supplementation; in the latter, not^22^ ^,^ ^23^. The diabetic patients in this sample did not have ulcers. A pressure ulcer was reconstructed in the sacral region, secondary to spinal cord trauma, which developed dehiscence due to osteomyelitis. It was not possible to treat venous ulcers, but one reconstruction of the lower limbs secondary to suppurative lymphangitis was performed, evolving without complications.

On the other hand, an uncontrolled inflammatory response can result in excessive production of type-III collagen, contributing to hypertrophic scars and keloids^24^. Van der Veer et al. found no significant difference in the incidence of hypertrophic scarring between groups that used Vitamin D and those that did not^23^. One patient with insufficient levels and one with sufficient ones had pathological scars, without the need for intervention.

With reduced collagen, the vessel wall may be weakened and hematomas may occur^15^ ^,^ ^24^ ^,^ ^25^. Only one hematoma was reported in an patient with insufficiency, without the need for intervention. Since the absorption and drainage of interstitial fluids is affected, there may be deficient blood perfusion in critical areas such as grafts and flaps, increasing the risk of ischemia and tissue necrosis^26^. There were no seromas in the sample.

Plastic surgeries have relatively lower rates of infection when compared with surgical procedures in other specialties. Most procedures are elective, which allows for a careful selection of healthy candidates or those with controlled comorbidities. Strict protocols of asepsis, antisepsis, hygiene, and antibiotic prophylaxis contribute to lower infection rates when compared with emergency procedures^27^. When a continuity solution occurs in the mechanical barrier of the skin, the innate immune system increases the expression of the toll-like-2 receptor and transforming growth factor, which activate Vitamin D in calcitriol. It induces the formation of protective peptides, such as catelecidin and defensins, which suppress pro-inflammatory cytokines and affect keratinocyte proliferation and migration and angiogenesis^1^ ^,^ ^22^ ^,^ ^28^ ^,^ ^29^. In a situation of Vitamin D deficiency, the expression of these peptides can be reduced, weakening the antimicrobial barrier of the skin and mucous membranes. This may increase the risk of bacterial colonization in surgical incisions, predisposing the patient to infections^30^. There were no patients being treated for burns in the study, but Chen et al. conducted a retrospective cohort study with this population and reported a significant reduction in length of hospital stay, incidence of wound infection, and septicemia in those who received daily supplementation of calcium, magnesium, and vitamins A, B1, B6, B12, C, D, and E^4^ ^,^ ^23^. In this study, infection ensued in one patient with insufficient level, without the need for intervention, and in one with sufficient level, requiring antibiotic therapy.

Vitamin D metabolites have a significant clinical role due to their interrelationship with calcium homeostasis, bone metabolism, and neuronal integrity. Deficient levels may contribute to the development of osteoporosis and increased risk of falls and fractures in the elderly^6^. Its maintenance may play a role in the postoperative period of plastic surgeries involving compact or spongy bone grafts. There were no such procedures in our sample. Bone surgeries, specifically of the spine, femur, or ankle have also not shown a direct relationship between Vitamin D levels and improved pain or quality of life^8^ ^,^ ^14^ ^,^ ^31^. Soares et al. suggested that hypocalcemia in thyroidectomized patients could be due to hypovitaminosis D. In fact, low serum calcium affects neuron excitability and muscle contraction, clinically translated as paresis. However, factors such as surgical technique, duration of anesthesia, and the patient’s individual characteristics should be considered in the preoperative evaluation of Vitamin D levels before attributing direct relationships to the risk of neuromuscular complications^17^. One deficient patient developed paresis after rhytidoplasty, requiring surgical reapproach. According to Zwiebel et al., patients undergoing facial plastic surgery seem to make more use of vitamin supplements, especially Vitamin D, compared with the general population^32^.

Low Vitamin D levels have also been associated with oral health problems, such as gum inflammation, tooth loss, loss of clinical attachment, and maternal periodontal disease during pregnancy^33^. Such events were not reported in this sample.

Hypovitaminosis D has been associated with significant increases in length of stay in intensive care units, treatment costs, and mortality rates. Previous studies have highlighted the prevalence of Vitamin D insufficiency in critically ill patients, which can be as high as 50%, with undetectable levels in up to 17% of cases. In addition, Vitamin D deficiency was a significant predictor of short- and long-term mortality, as well as positivity in blood cultures^10^ ^,^ ^34^. During the COVID-19 pandemic, Evans et al. discussed the use of paricalcitol, a synthetic analogue, as a strategy to improve the immune response and reduce the need for mechanical ventilation. There were no patients admitted to the intensive care unit in their sample, but it should be considered that sun exposure was reduced due to the period of social isolation^35^.

The main sources of Vitamin D are skin synthesis and foods such as fatty fish. The two commonly available forms of supplements are cholecalciferol, or Vitamin D3, and ergocalciferol, or Vitamin D2. These are not regulated by the United States Food and Drug Administration (FDA), which leads to lack of data on safety and efficacy. In any case, in a meta-analysis of seven randomized controlled trials evaluating serum 25(OH)D concentrations after supplementation with both presentations, cholecalciferol increased serum 25(OH)D concentrations more efficiently than ergocalciferol. However, the trials used varying doses and treatment periods, resulting in significant heterogeneity between studies^9^. In cases of adults with deficiency, initial doses of 6,000 IU daily or 15,000 IU weekly for eight weeks are recommended, followed by maintenance with 1,500 to 2,000 IU daily^8^ ^,^ ^36^. Pre or postoperative supplementation was not prescribed for the patients in this study.

This study can be considered a pilot study, as there are some limitations. The heterogeneity of a small population and the broad spectrum of reconstructive and aesthetic surgeries did not allow us to demonstrate the possible deleterious effect of Vitamin D insufficiency or deficiency on surgical outcomes. Future research should focus on specific demographic subgroups and procedures.

## CONCLUSION

Preoperative hypovitaminosis D has not been associated with complications in plastic surgery.
